# Sarco/endoplasmic reticulum calcium ATPase activity is unchanged despite increased myofilament calcium sensitivity in Zucker type 2 diabetic fatty rat heart

**DOI:** 10.1038/s41598-022-20520-0

**Published:** 2022-10-07

**Authors:** Yann Huey Ng, Regis R. Lamberts, Peter P. Jones, Ivan A. Sammut, Gary M. Diffee, Gerard T. Wilkins, James C. Baldi

**Affiliations:** 1 Department of Medicine and HeartOtago, Otago Medical School, University of Otago, 201 Great King Street, Dunedin Central, Dunedin, 9016 New Zealand; 2 Department of Physiology and HeartOtago, School of Biomedical Sciences, University of Otago, Dunedin, New Zealand; 3 Department of Pharmacology and Toxicology and HeartOtago, School of Biomedical Sciences, University of Otago, Dunedin, New Zealand; 4 Department of Kinesiology, University of Wisconsin-Madison, Madison, WI USA

**Keywords:** Calcium signalling, Cardiovascular diseases, Post-translational modifications, Cell biology

## Abstract

Systolic and diastolic dysfunction in diabetes have frequently been associated with abnormal calcium (Ca^2+^) regulation. However, there is emerging evidence that Ca^2+^ mishandling alone is insufficient to fully explain diabetic heart dysfunction, with focus shifting to the properties of the myofilament proteins. Our aim was to examine the effects of diabetes on myofilament Ca^2+^ sensitivity and Ca^2+^ handling in left ventricular tissues isolated from the same type 2 diabetic rat hearts. We measured the force-pCa relationship in skinned left ventricular cardiomyocytes isolated from 20-week-old type 2 diabetic and non-diabetic rats. Myofilament Ca^2+^ sensitivity was greater in the diabetic relative to non-diabetic cardiomyocytes, and this corresponded with lower phosphorylation of cardiac troponin I (cTnI) at ser23/24 in the diabetic left ventricular tissues. Protein expression of sarco/endoplasmic reticulum Ca^2+^-ATPase (SERCA), phosphorylation of phospholamban (PLB) at Ser16, and SERCA/PLB ratio were lower in the diabetic left ventricular tissues. However, the maximum SERCA Ca^2+^ uptake rate was not different between the diabetic and non-diabetic myocardium. Our data suggest that impaired contractility in the diabetic heart is not caused by SERCA Ca^2+^ mishandling. This study highlights the important role of the cardiac myofilament and provides new insight on the pathophysiology of diabetic heart dysfunction.

## Introduction

Diabetes impairs heart function^1^, and is associated with increased rates of heart failure^2^. Our group^3–5^ and others^6,7^ have found that contractility is impaired in type 2 diabetic hearts, in advance of heart failure development. Decades of research has suggested that abnormal calcium (Ca^2+^) flux impairs cardiac contractility and relaxation in the type 2 diabetic heart^8,9^. For example, cardiomyocytes from type 2 diabetic hearts with depressed cardiac function have decreased Ca^2+^ flux during systole and diastole, reduced Ca^2+^ transient decay rate, and increased sarcoplasmic reticulum Ca^2+^ leakage^9,10^. Sarco/endoplasmic reticulum Ca^2+^ ATPase (SERCA) is the major Ca^2+^ pump regulating cytoplasmic Ca^2+^ flux, and defects in SERCA have frequently been associated with impaired cardiac function in diabetes^9,11,12^. SERCA expression is upregulated in the right atria^13^, but unchanged in the left ventricular tissues^14^ obtained from type 2 diabetic patients with impaired diastolic function. However, protein expression does not always reflect the intrinsic activity of SERCA. Vetter et al*.* found that increased SERCA Ca^2+^ uptake improved myocardial contractility in type 1 diabetic rat hearts^15^, suggesting that reduced SERCA activity contributed to cardiac dysfunction in diabetes; presumably by deranging cytosolic Ca^2+^ flux.

However, others have reported that SERCA activity is *increased* in type 2 diabetic hearts^16,17^, and that intracellular Ca^2+^ transient amplitudes are unaffected by diabetes^18,19^. Misra et al*.* found that SERCA Ca^2+^ uptake rate was increased in type 2 diabetic cp/cp rat hearts, but intracellular Ca^2+^ concentration was not different at basal conditions^17^. Daniels et al*.* also recently found that cardiac function could be restored in diabetic rat trabeculae by kinase inhibition without any change in Ca^2+^ flux^20^. These findings suggest that SERCA alone does not regulate the intracellular Ca^2+^ state, and that altered intracellular Ca^2+^ regulation is not the only factor causing contractile dysfunction in diabetic hearts.

An alternative explanation for impaired contractility is that the Ca^2+^ sensitivity of cardiac myofilaments is altered in the type 2 diabetic heart. The Ca^2+^ sensitivity of the myofilament is affected by transient post-translational modifications of myofilament proteins^21^, particularly cardiac troponin I (cTnI)^22,23^. In both type 1 and type 2 diabetic animal models, myofilament Ca^2+^ sensitivity of cardiomyocytes has been reported as unaltered^6,24^, increased^25^ or decreased^26^. The obese Zucker diabetic fatty (ZDF) rat is a type 2 diabetic model with well-established cardiac inotropic and lusitropic dysfunction^3,6,27^. Our group recently found that myofilament Ca^2+^ sensitivity was increased in 20-week-old type 2 diabetic ZDF rat cardiomyocytes, and this was associated with reduced phosphorylation of cTnI at Ser23/24^28^. Interestingly, increased myofilament Ca^2+^ sensitivity would be expected to enhance cardiac contractility by increasing the affinity of thick and thin filament interaction^29^. Therefore, it is unclear why contractile dysfunction still existed in these hearts. One possibility is that deranged SERCA activity reduces intracellular Ca^2+^ concentrations. Reduced SERCA Ca^2+^ uptake has been associated with reduced systole Ca^2+^ transient amplitude and impaired cardiac contractility in type 2 diabetic mouse heart^11^. Reduced SERCA-mediated Ca^2+^ uptake activity may decrease sarcoplasmic reticulum Ca^2+^ content, hence less intracellular Ca^2+^ would be available for the subsequent contractions, and this may explain systolic dysfunction in the diabetic heart.

As our group has recently found that myofilament Ca^2+^ sensitivity is increased in type 2 diabetic heart^28^ which is not normally associated with contractile dysfunction, we therefore hypothesised that SERCA expression and activity would be reduced in 20-week-old type 2 diabetic rat heart.

## Results

### Animal characteristics

Non-fasting blood glucose was higher in the diabetic versus non-diabetic rats (30.6 ± 0.9 mmol/L vs. 11.9 ± 0.5 mmol/L, respectively, *P* < 0.0001), confirming their diabetic status, as previously reported^3,27^. However, body weight was not different between the diabetic and non-diabetic rats (403.0 ± 17.3 g vs. 395.4 ± 15.2 g, respectively, *P* = 0.75).

### Cardiomyocyte myofilament Ca^2+^ sensitivity

**Figure 1:**
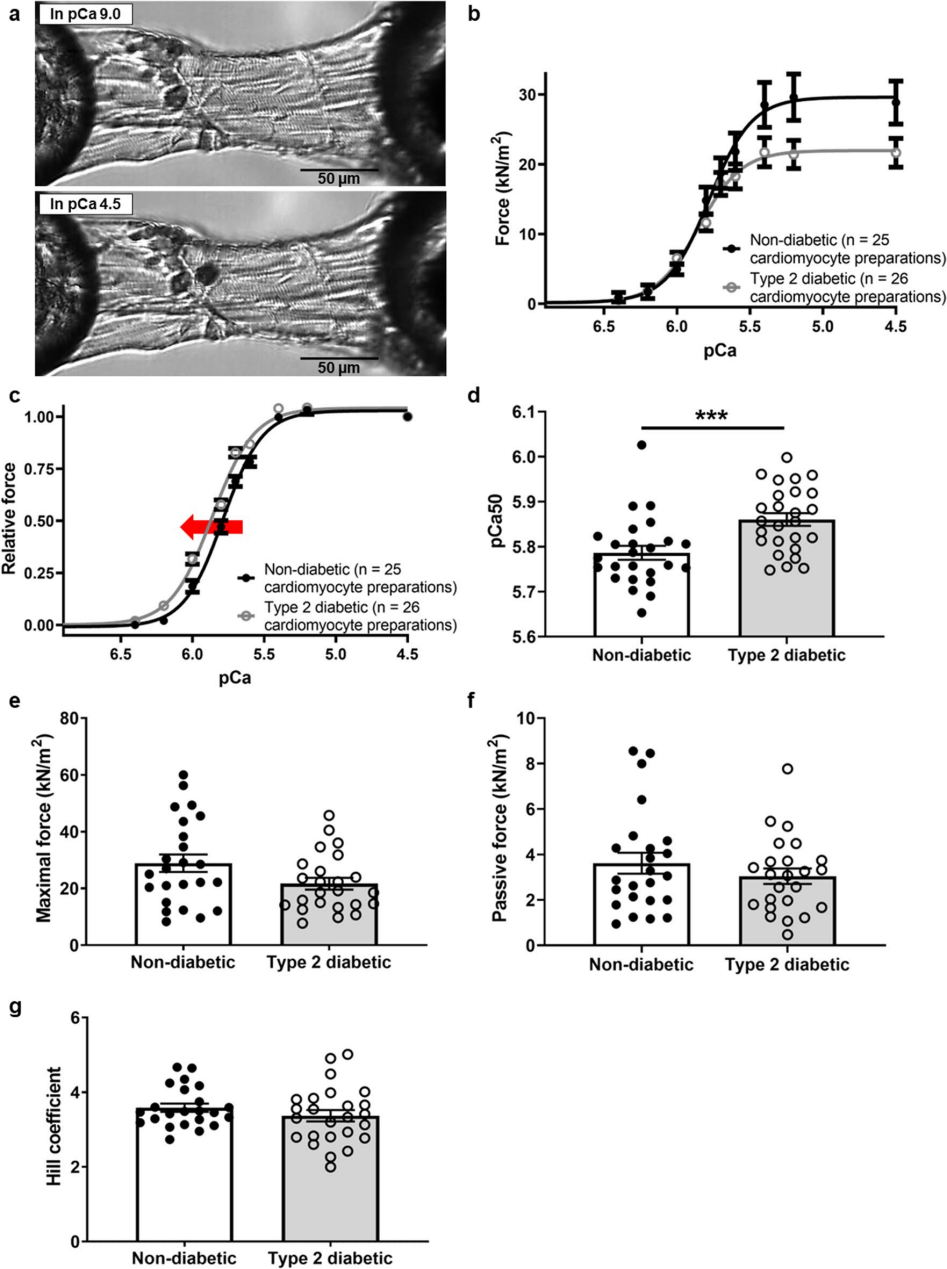
Force-pCa measurements on skinned rat cardiomyocyte preparations. (**a**) A representative photomicrograph of a 10x-magnified skinned rat cardiomyocyte preparation mounted on the experimental apparatus and bathed in relaxing solution pCa 9.0 and maximally activating solution pCa 4.5. (**b**) Absolute force-pCa curves of non-diabetic (n = 5 animals) and diabetic (n = 5 animals) cardiomyocyte preparations. (**c**) Average pooled relative force-pCa relationships of non-diabetic and diabetic cardiomyocyte preparations at a sarcomere length of 2.2 µm. The red arrow indicates a leftward shift of the force-pCa curve in the diabetic cardiomyocyte preparations. (**d**) pCa50, (**e**) maximal force per cross sectional area, (**f**) passive force per cross sectional area, and (**g**) Hill coefficient of individual left ventricular skinned cardiomyocyte preparations isolated from the non-diabetic and type 2 diabetic rat hearts. Data are presented as means ± SEM, ***P < 0.001 versus non-diabetics, unpaired t-test.

A representative image of a skinned rat cardiomyocyte preparation mounted on the experimental apparatus is shown in Fig. 1a. The absolute force-pCa50 curves for diabetic and non-diabetic cardiomyocyte preparations are shown in Fig. 1b. The relative force-pCa50 curve was shifted to the left in the diabetic hearts (Fig. 1c). As a result, the pCa50 was greater in the diabetic versus non-diabetic hearts (5.86 ± 0.01 vs. 5.79 ± 0.02, respectively, *P* = 0.0009, Fig. 1d). There were no differences in maximal force per cross-sectional area (diabetic 21.65 ± 2.07 kN/m^2^ vs. non-diabetic 28.84 ± 3.09 kN/m^2^, *P* = 0.06, Fig. 1e), passive force per cross-sectional area (diabetic 3.04 ± 0.34 kN/m^2^ vs. non-diabetic 3.61 ± 0.46 kN/m^2^, *P* = 0.32, Fig. 1f), or the Hill coefficient (diabetic 3.37 ± 0.15 vs. non-diabetic 3.59 ± 0.11 kN/m^2^, *P* = 0.26, Fig. 1g) between groups.

### Protein expression

**Figure 2:**
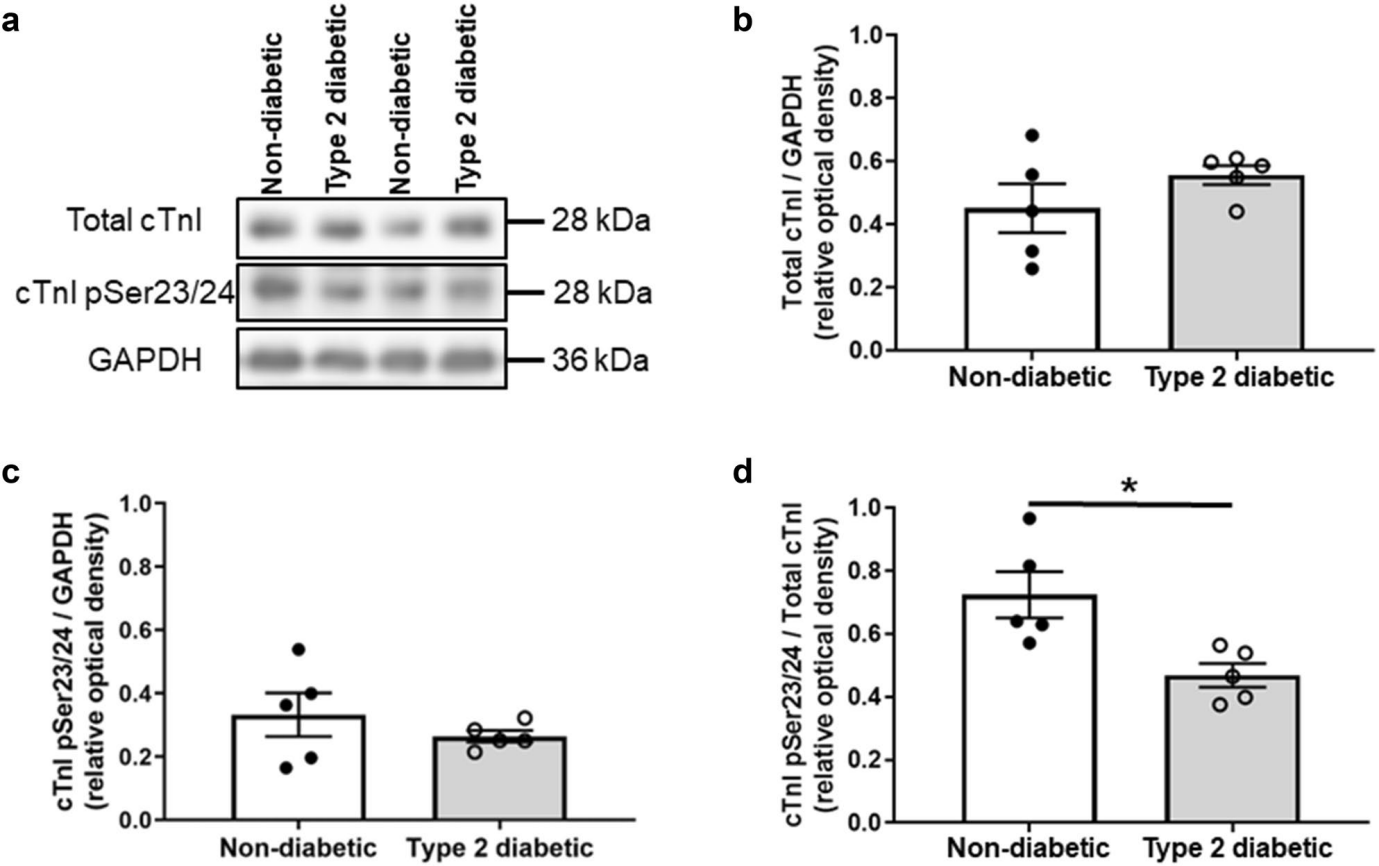
Protein expression of total cTnI and phosphorylation of cTnI at Ser23/24 in non-diabetic and diabetic rat left ventricular tissues. (**a**) Representative immunoblots of total cTnI, phosphorylated cTnI Ser23/24 (cTnI pSer23/24), and GAPDH in non-diabetic and diabetic rat left ventricular tissues. Quantification of (**b**) total cTnI/GAPDH, (**c**) phosphorylated cTnI Ser23/24 / GAPDH, and (**d**) phosphorylated cTnI Ser23/24/total cTnI levels in non-diabetic and diabetic rat left ventricular tissues. Data shown are means ± SEM, obtained from duplicate blots, *P < 0.05 versus non-diabetics, unpaired t-test. Original immunoblots are presented in Supplementary Fig S1.

The total cTnI expression was not different in the left ventricular tissue isolated from the diabetic and non-diabetic hearts (0.56 ± 0.03 a.u. vs. 0.45 ± 0.08 a.u., respectively, *P* = 0.24, Fig. 2a,b). Phosphorylation of cTnI at Ser23/24 normalised to GAPDH was not different between groups (0.26 ± 0.02 a.u. in diabetics vs. 0.33 ± 0.07 a.u. in non-diabetics, *P* = 0.37, Fig. 2c). However, the ratio of phosphorylation of cTnI at Ser23/24 against the total cTnI was lower in the diabetic compared to the non-diabetic hearts (0.47 ± 0.04 a.u. vs. 0.72 ± 0.07 a.u., respectively, *P* = 0.014 Fig. 2d).

**Figure 3:**
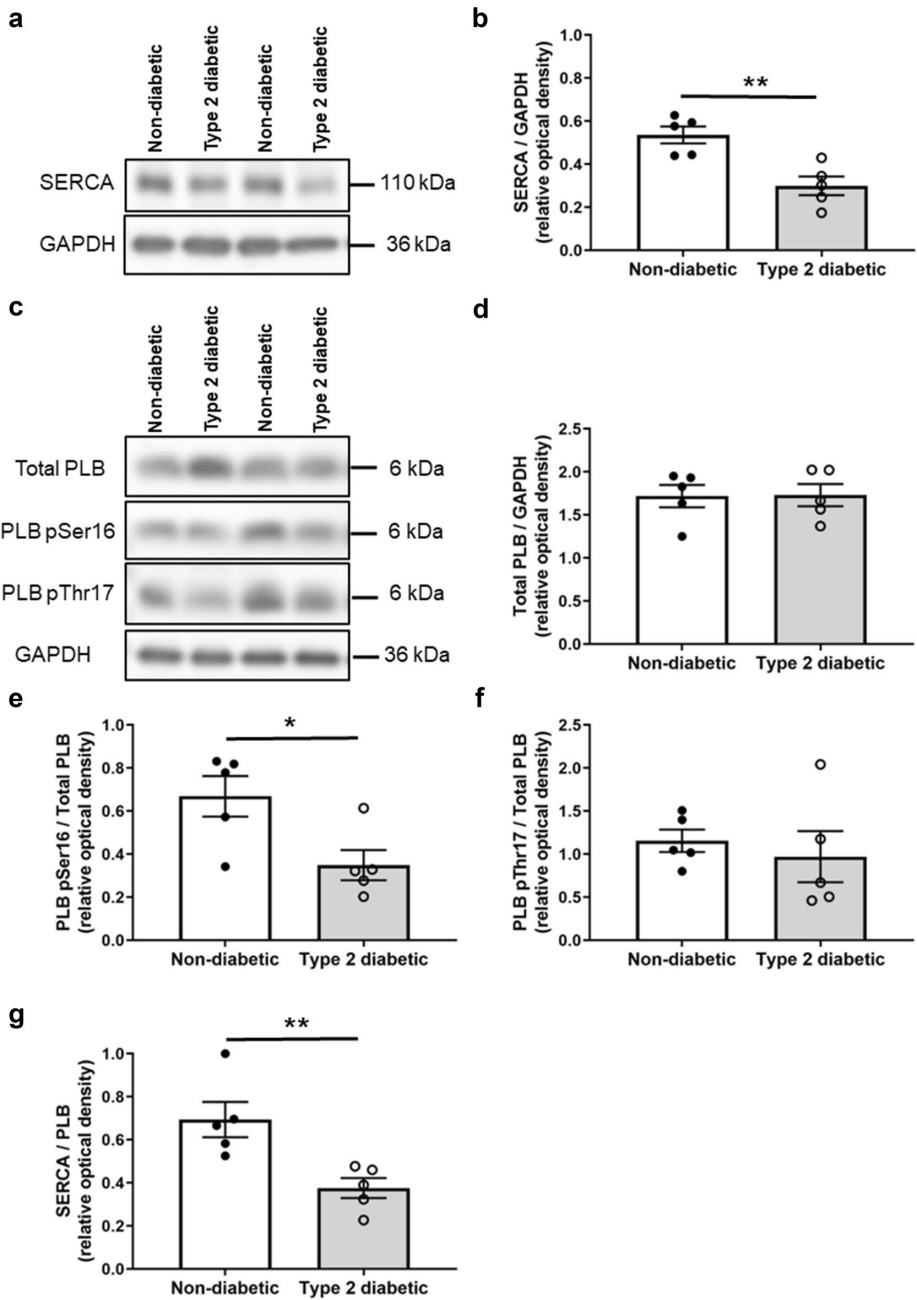
Protein expression of SERCA, phospholamban, and phosphorylated phospholamban at Ser16 and Thr17 in non-diabetic and diabetic rat left ventricular tissues. (**a**) Representative immunoblots of SERCA and GAPDH. (**b**) Quantification of SERCA level in non-diabetic and diabetic hearts. (**c**) Representative immunoblots of total PLB, phosphorylated PLB at Ser16 (PLB pSer16) and Thr17 (PLB pThr17), and GAPDH as a loading control. Quantification of (**d**) total PLB levels, (**e**) phosphorylated PLB Ser16, and (**f**) phosphorylated PLB Thr17 levels in non-diabetic and diabetic hearts. (**g**) SERCA/PLB ratio in non-diabetic and diabetic hearts. Data shown are means ± SEM, obtained from duplicate blots, *P < 0.05, **P < 0.01 versus non-diabetics, unpaired t-test. Original immunoblots are presented in Supplementary Fig S1.

**Figure 4:**
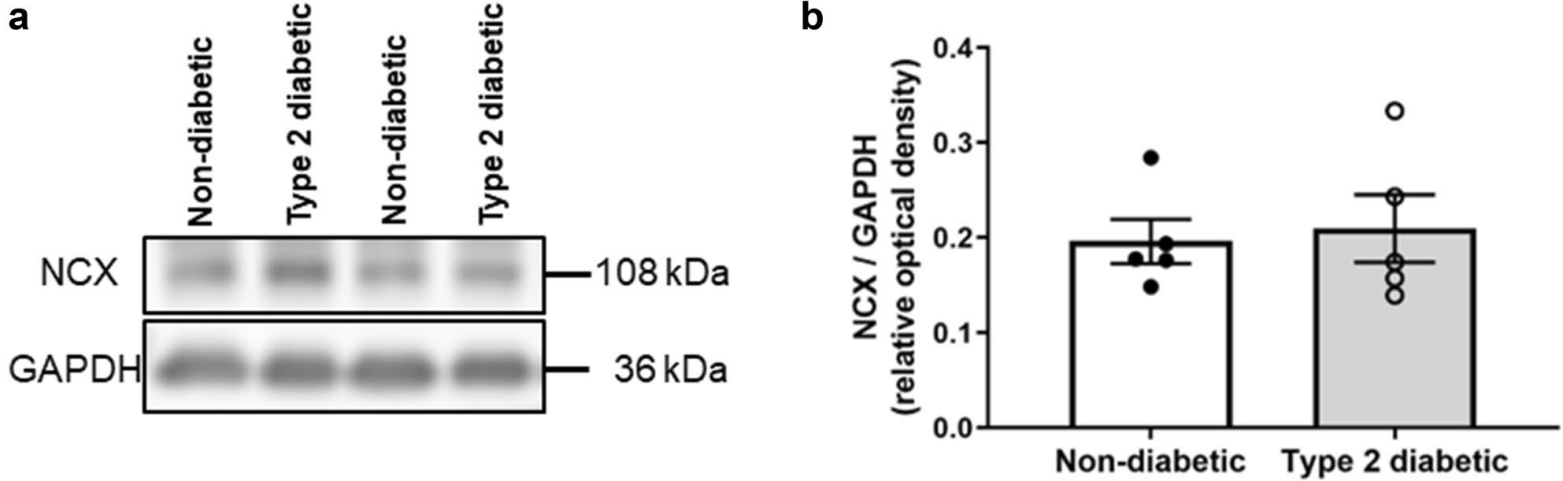
Protein expression of NCX in non-diabetic and diabetic rat left ventricular tissues. (**a**) Representative immunoblots of NCX and GAPDH. (**b**) Quantification of NCX levels in non-diabetic and diabetic hearts. Data shown are means ± SEM, obtained from duplicate blots, *P* > 0.05 versus non-diabetics, unpaired t-test. Original immunoblots are presented in Supplementary Fig S1.

SERCA expression was lower in the diabetic hearts when compared to the non-diabetic controls (0.30 ± 0.04 a.u. vs. 0.54 ± 0.04 a.u., respectively, *P* = 0.0037, Fig. 3a,b). The total PLB expression was not different between the diabetic and non-diabetic hearts (1.73 ± 0.13 a.u. vs. 1.72 ± 0.13 a.u., respectively, *P* = 0.96, Fig. 3c,d). The resultant ratio of SERCA/PLB was lower in the diabetic hearts (0.38 ± 0.05 a.u. vs. 0.69 ± 0.08 a.u. in the non-diabetics, *P* = 0.0096, Fig. 3g). Phosphorylation of PLB at Ser16 was also lower in the diabetic hearts (0.35 ± 0.07 a.u. vs. 0.67 ± 0.09 a.u. in the non-diabetics, *P* = 0.026, Fig. 3e); whereas phosphorylation of PLB at Thr17 was not different between groups (0.97 ± 0.03 a.u. in the diabetics vs. 1.15 ± 0.13 a.u. in the non-diabetics, *P* = 0.59, Fig. 3f). No difference was found in NCX expression between the diabetic and non-diabetic hearts (0.21 ± 0.04 a.u. vs. 0.20 ± 0.02 a.u., respectively, *P* = 0.76, Fig. 4a,b).

### SERCA Ca^2+^ uptake activity

**Figure 5:**
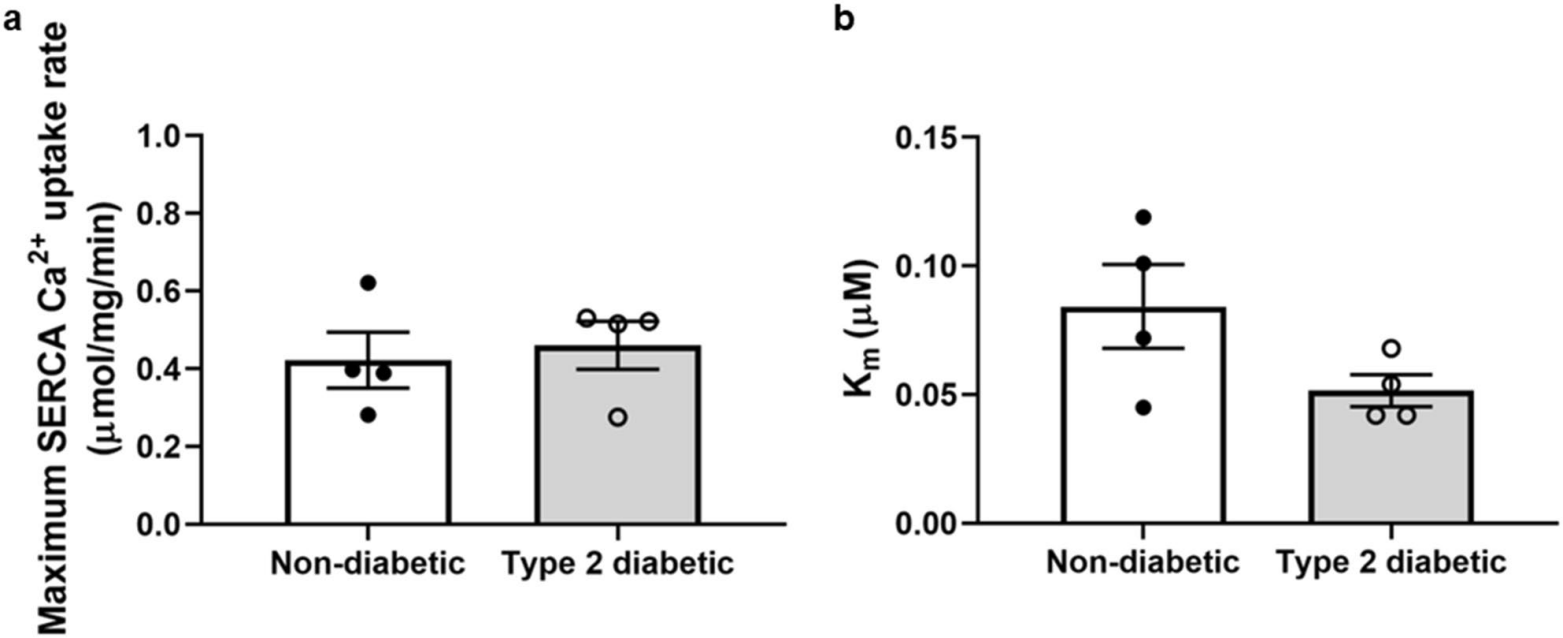
SERCA activity in non-diabetic and diabetic rat myocardium. (**a**) The maximum SERCA Ca^2+^ uptake rate, and (**b**) Michaelis constant (K_m_) for the non-diabetic and diabetic rat myocardium. Data shown are means ± SEM, obtained from triplicate readings, *P* > 0.05 versus non-diabetics, unpaired t-test.

The maximum SERCA Ca^2+^ uptake rate was not different between the diabetic and non-diabetic hearts (0.46 ± 0.06 µmol/mg/min vs. 0.42 ± 0.07 µmol/mg/min, respectively, *P* = 0.70, Fig. 5a). Similarly, the Michaelis constant (K_m_) was not different between the diabetic and non-diabetic hearts (0.052 ± 0.006 µM vs. 0.084 ± 0.016 µM, respectively, *P* = 0.11, Fig. 5b).

## Discussion

The obese diabetic ZDF rat heart is characterised by depressed contractility but *increased* myofilament Ca^2+^ sensitivity^28^. This implies that reduced Ca^2+^ availability exists during systole in order to account for reduced force generation. In this context, we hypothesised that the activity of SERCA, the protein most responsible for the intracellular Ca^2+^ flux, would be lower in the obese diabetic ZDF rat left ventricle. However, SERCA activity was not different between groups, despite the fact that the SERCA/PLB ratio was lower in the diabetic hearts. Similarly, the expression of NCX, which extrudes Ca^2+^ from the cardiomyocyte, was not different between groups. As previously shown^28^, we found that myofilament Ca^2+^ sensitivity was greater, and that this augmentation was associated with lower phosphorylation of cTnI at Ser23/24 in the diabetic hearts compared to the non-diabetic hearts. Therefore, while left ventricular contractility is reduced in this diabetic heart model, our findings suggest this dysfunction is not caused by changes in SERCA activity.

Increased myofilament Ca^2+^ sensitivity in the obese diabetic ZDF rat cardiomyocyte suggests that *less* Ca^2+^ would be required to elicit a given contractile force than in the non-diabetic cardiomyocytes. Numerous isolated heart experiments have shown that left ventricular force is reduced in this model^3,27^; which when combined with increased Ca^2+^ sensitivity, implies that the intracellular Ca^2+^ concentration in diabetic cardiomyocytes is reduced; even in the absence of any difference in SERCA activity. Indeed, Belke et al*.* found that intracellular Ca^2+^ *concentration* during systole and diastole was significantly lower in type 2 diabetic *db/db* mouse isolated myocytes^9^, which they attributed to decreased Ca^2+^ handling by the sarcoplasmic reticulum. However, Zhang et al*.* argued that reduced expression of SERCA could explain prolonged action potentials and slower Ca^2+^ transients, but not contractile deficits; which they attributed to remodelling of the myofilament, specifically a reduction in actin content^18^. Others have reported that reduced NCX activity impairs contractile function in type 1 diabetic rat models^30,31^. We did not quantify intracellular Ca^2+^ transient amplitude in our experiments, but found no evidence to suggest that deranged Ca^2+^ handling by either SERCA or NCX was associated with poor contractility in our experiment. Nonetheless, the combination of decreased contractility of isolated hearts and increased myofilament Ca^2+^ sensitivity support the idea that intracellular Ca^2+^ concentration may have been lower in the diabetic myocardium.

Although the protein expression of SERCA and PLB have been used as indicators of SERCA activity, our data, and others^12,32^ suggest that protein levels do not necessarily reflect the intrinsic SERCA activity. We found that SERCA/PLB ratio and phosphorylated PLB at Ser16 were lower in the diabetic hearts, without any difference in the maximum SERCA Ca^2+^ uptake rate between groups. The discrepancy between SERCA expression and activity may be explained by the ‘coupling’ between SERCA and PLB. Several studies have shown that only a fraction of SERCA is functionally regulated by PLB in the heart^33,34^. Brittsan et al*.* found that SERCA Ca^2+^ uptake rate was proportionately reduced with increasing PLB expression in transgenic mouse model, and only ~ 40% of SERCA was under the inhibitory regulation of PLB in murine hearts under basal conditions^34^. This suggests that the ‘coupling’ between SERCA and PLB may not be affected by diabetes in the obese diabetic ZDF rat myocardium and this may explain why SERCA Ca^2+^ uptake activity was unaltered despite a lower SERCA/PLB ratio in the diabetic left ventricular tissues.

Our finding that the diabetic ZDF rat heart had greater myofilament Ca^2+^ sensitivity and lower phosphorylation of cTnI at Ser23/24^28^ reinforces the inverse association between myofilament Ca^2+^ sensitivity and phosphorylation of cTnI at Ser23/24 described in heart disease^21,35^. Similarly, our data show that phosphorylation of PLB at Ser16 was lower in the diabetic rat left ventricular tissues. Phosphorylation of cTnI at Ser23/24 and PLB at Ser16 are commonly recognised as PKA-dependent phosphorylation sites mediated by the cyclic adenosine monophosphate (cAMP) pathway^21,36^. Bockus and Humphries found that inotropic and lusitropic responses were impaired in type 1 diabetic mice following acute activation of PKA^37^. Thaung et al*.* also found that the expression of G_s_ protein, which increases cAMP level during β-adrenergic stimulation, was reduced by 28% in this type 2 diabetic ZDF rat model^27^. Therefore, our data suggest that cAMP/PKA signalling may be impaired in the diabetic heart. Interestingly, Daniels et al*.* found that contractility and relaxation were restored in diabetic rat trabeculae without any change in sarcoplasmic reticulum Ca^2+^ flux when Ca^2+^/calmodulin dependent kinase II (CaMKII) was inhibited^20^. Unfortunately, the authors did not determine whether this effect was associated with alterations in the phosphorylation of cTnI or other myofilament proteins known to alter Ca^2+^ sensitivity.

Though not the focus of our study, the functional consequences of increased myofilament Ca^2+^ sensitivity may be involved in diabetic diastolic dysfunction. Increased baseline systole^38^, prolonged systolic duration^39^ and slower diastolic relaxation^13^ have been identified as early manifestations of diabetes, which precede cardiovascular disease. The greater myofilament Ca^2+^ sensitivity in the diabetic rat hearts in this study may suggest stronger binding of Ca^2+^ ions to cardiac troponin C (cTnC), which would delay or reduce the rate of Ca^2+^ dissociation from cTnC^40,41^. This increased affinity of Ca^2+^ for the troponin complex would prolong thick and thin filament interactions, thereby ‘delaying’ the onset of relaxation, leading to incomplete cardiac relaxation as often described in the diabetic heart^27^.

Our data may indicate that diabetes impacts the force generating components of the myofilament itself. It is possible that diabetes reduced cardiomyocyte force generation by interfering with the Ca^2+^-myofilament interaction. We found that skinned diabetic cardiomyocytes tended to produce less maximal force (~ 25%, *P* = 0.06) when bathed in supra-physiological Ca^2+^ (pCa 4.5). In such conditions, it is assumed that all thick and thin filament cross-bridges are activated, and any difference in force is explained by differences in the quantity of force producing cross-bridges per unit area. Jweied et al*.* similarly found that maximal force was 29% lower in diabetic human skinned cardiomyocytes (*P* = 0.08 vs. non-diabetic controls)^42^ and van den Brom et al*.* found that maximal force was 68% lower in 14-week-old type 2 diabetic ZDF rats with impaired cardiac function^6^. Zhang et al*.* found that the area of the actin filament was 8% lower in streptozotocin-induced diabetic rat myofilaments, and that the sarcomeric organization of actin was disorganized compared to non-diabetic myofilaments^18^. A reduction in the content or quality of force generating proteins in the diabetic left ventricular cardiomyocyte would explain the consistent finding that maximal force is reduced in isolated heart experiments in this model^3,6,27^. It is also possible that the increase in myofilament Ca^2+^ sensitivity would compensate for this loss of generated force by eliciting greater contractile activation at lower intracellular Ca^2+^ concentrations.

Previous studies by our group have consistently shown that type 2 diabetic ZDF rats have impaired cardiac contractility^20,27^ and coincide with *increased* myofilament Ca^2+^ sensitivity^28^. SERCA is the primary Ca^2+^ pump in cardiomyocytes; however, the lack of change in SERCA activity may indicate that an alternative Ca^2+^ transporter is altered by diabetes. Several studies have suggested that NCX activity is increased in diabetic cardiomyocytes^8,11^, and may ‘compete’ with SERCA to remove Ca^2+^ from the cytosol. We did not measure NCX activity in the present study. However, NCX expression was not different between the diabetic and non-diabetic myocardium, which may indicate a normal Ca^2+^ efflux under basal condition. Similarly, Belke et al*.* found that NCX activity was not different between type 2 diabetic and non-diabetic hearts^9^. A more exhaustive comparison of the various Ca^2+^ ion channels (i.e. L-type, NCX, ryanodine receptors, SERCA), and the dynamic interactions between Ca^2+^ and the myofilaments may be necessary to better understand the mechanism of diabetic heart dysfunction.

Our data show that the type 2 diabetic left ventricular cardiomyocytes have greater myofilament Ca^2+^ sensitivity and lower phosphorylation of cTnI at Ser23/24. SERCA/PLB ratio was reduced, however, SERCA Ca^2+^ uptake activity was unaltered in the diabetic hearts, suggesting that impaired contractility may be associated with changes in intracellular Ca^2+^ concentration independent of SERCA. These findings suggest an important role for myofilaments and generate new questions regarding the pathophysiological mechanism in diabetic heart dysfunction.

## Methods

### Animals

Experiments were conducted on 20-week-old male type 2 diabetic Zucker Diabetic Fatty (ZDF^fa/fa^; n = 5) rats and their non-diabetic (homozygous ZDF^+/+^ or heterozygous ZDF^+/-^; n = 5) littermates. Animals were obtained from an in-house colony, with the original breeding pairs obtained from Charles River Laboratories (Wilmington, MA, USA). Animals were housed in groups of 2 per cage with a standard 12-h light–dark cycle (0700 – 1900) and stable temperature of 21 ± 1 °C. Animals were fed with rat chow Purina 5008 (LABDIET) and had access to food and water ad libitum.

At 20 weeks of age, the animals were weighed and deeply anaesthetised with 60 mg/kg sodium pentobarbital via intraperitoneal injection. When the pedal withdrawal reflex was absent, the chest was cut open, the heart was quickly excised and rinsed in ice-cold Krebs–Henseleit buffer. The left ventricle was cut into several pieces, flash frozen in liquid nitrogen and stored at -80 °C. A thorax blood plasma glucose test was performed using a glucometer and test strips (ACCU-CHEK Performa, Roche) to confirm diabetic status.

### Cardiomyocyte force-pCa measurements

Force measurements were performed on isolated skinned cardiomyocyte preparations at 15 °C as described by Diffee and Nagle^43^. The left ventricular tissues from the different groups were blinded and randomised to eliminate bias. A unique identification number was assigned to the left ventricular tissue of each animal and kept until all the measurements were completed. Briefly, left ventricular samples were defrosted in relaxing solution containing the following (mM): 100 KCl, 1.75 EGTA, 10 imidazole, 4 ATP-Mg, and 5 MgCl_2_; pH 7.0. The left ventricular tissue was mechanically disrupted and incubated in relaxing solution supplemented with 1% Triton X-100 for 8 min to permeabilise the sarcolemmal membranes. The resulting isolated, skinned cardiomyocyte preparations were washed twice in relaxing solution. Thereafter, a skinned cardiomyocyte preparation (1–3 cells) was attached between a force transducer and a piezoelectric motor with silicon adhesive. Sarcomere length of the skinned cardiomyocyte preparation was adjusted to approximately 2.20 µm for isometric force measurements. The force was measured as a function of pCa (− log_10_[Ca^2+^]) in solutions ranging from pCa 9.0 (relaxing solution) to pCa 4.5 (maximally activating solution).

Force was first measured in pCa 9.0 and 4.5 solutions and then in randomly ordered submaximal pCa solutions, followed by another measurement made in pCa 9.0 and 4.5 to assess any decline in cardiomyocyte performance. Measurements were continued in another three to four randomly ordered submaximal pCa solutions until a full force-pCa sigmoidal curve was obtained. This was followed by a last control measurement in pCa 9.0 and 4.5 to assess force rundown in the cardiomyocyte preparations. Skinned cardiomyocyte preparations without clear striations for sarcomere length measurement and that did not maintain more than 80% of maximal force at the end of the experimental protocol were discarded and data not used. For each activation, when steady-state force was reached, force was measured by abruptly slackened the skinned cardiomyocyte preparation by 20% of its initial length and transferred to pCa 9.0. Total force was calculated as the difference between steady-state force and the baseline force immediately after the skinned cardiomyocyte preparation was slacked. Active force was determined by subtracting passive force in pCa 9.0 from total force. Maximal force (force developed in pCa 4.5) and passive force (force developed in pCa 9.0) were normalised to the cross-sectional area (CSA) of the skinned cardiomyocyte preparation $({\text{CSA}} = 3.14 \times ({\text{cell}}\;{\text{width}}/2)^{2})$. Active force at each pCa was expressed as a percentage of maximal force for that skinned cardiomyocyte preparation. Force-pCa relationships for each skinned cardiomyocyte preparation were fit to a modified Hill equation.

$$\text{F}= {\text{F}}_{\text{max}} \times {\left[{\text{Ca}}^{2+}\right]}^{\text{nH}}/ ({\text{pCa}}_{50}^{\text{nH}}+ {\left[{\text{Ca}}^{2+}\right]}^{\text{nH}})$$

where ${\text{F}}$ is steady state force, ${\text{F}}_{\text{max}}$ is the maximum isometric force at saturating Ca^2+^ concentration, ${\text{nH}}$ (Hill coefficient) is a measure of the steepness of the force-pCa relation which is an index of myofilament cooperative activation, ${\text{pCa}}_{50}$ is Ca^2+^ concentration that produced 50% of maximal force which is used as an index of myofilament Ca^2+^ sensitivity.

### SERCA Ca^2+^ uptake activity assay

Left ventricular tissue (~ 0.3 g) was mechanically disrupted in sarcoplasmic reticulum isolation buffer, which contained (mM): 290 sucrose, 10 imidazole, 3 NaN_3_, 0.5 PMSF, 0.5 DTT, 0.2 g/L trypsin inhibitor, and 0.2 g/L benzamide, pH 6.90. The tissue homogenate was centrifuged at 2300*g* for 15 min at 4 °C. The pellet was discarded and the supernatant was centrifuged at 50,000*g* for 3.5 h at 4 °C. After centrifugation, the supernatant was discarded and the pellet was re-suspended in 1 mL sarcoplasmic reticulum isolation buffer supplemented with 650 mM KCl, and incubated on ice for 45 min, followed by another centrifugation at 2300*g* for 10 min at 4 °C. The pellet was discarded and the supernatant was centrifuged at 50,000*g* for 4 h at 4 °C. The supernatant was discarded and the resulting pellet containing the isolated sarcoplasmic reticulum vesicles was re-suspended in isolation buffer, snap frozen in liquid nitrogen, and stored at − 80 °C for the SERCA Ca^2+^ uptake assay.

Ca^2+^ uptake into the cardiac sarcoplasmic reticulum vesicles was measured according to the assay described by Beca et al*.* with some modifications^44^. The assay was performed at 30 °C in a 96-well plate with 100 µg of vesicles in a final volume of 100 µL of uptake buffer per well containing (mM): 100 KCl, 4 MgCl_2_, 20 HEPES, and 10 sodium oxalate, pH 7.0. The starting Ca^2+^ concentration was adjusted to approximately 1.5 µM as assessed by the fluorescence ratio of fura-2. The reactions were initiated with a premix containing (mM): 1.5 Na_2_ATP, 1.5 creatine phosphate and 2 U/ml creatine phosphokinase. The fluorescence signals at 340 nm and 380 nm were recorded at 20-s intervals for 25 min with a multi-mode microplate reader (SpectraMax i3X, Molecular Devices). The rate of reaction for sarcoplasmic reticulum Ca^2+^ uptake corrected for the amount of vesicle protein was calculated using the following formula:

$${\text{Rate}}\,{\text{of}}\,{\text{reaction}} = \left( {{\text{Volume}}\,{\text{of}}\,{\text{well}}\,\left( {\upmu {\text{L}}} \right)/{\text{Weight}}\,{\text{of}}\,{\text{vesicle}}\,{\text{protein}}\,\left( {\upmu {\text{g}}} \right)} \right)\times \left( { - \Delta \left[ {{\text{Ca}}^{2 + } } \right]_{{{\text{Total}}}} / \, \Delta {\text{Time}}} \right)$$

The data were plotted as rate of reaction against concentration of free Ca^2+^ ([Ca^2+^]_free_), and the maximum Ca^2+^ uptake rate was estimated from the plot by fitting a curve to the Michaelis–Menten equation.

The same uptake buffer was used for both diabetic and non-diabetic preparations, and the assay for both groups was run in parallel to minimise any potential variability in terms of the presence of heavy metals and Ca^2+^ buffering capacity. In a separate experiment, thapsigargin, a potent SERCA inhibitor^45^ completely inhibited Ca^2+^ uptake in isolated sarcoplasmic reticulum preparation (see Supplementary Fig S2), confirming that the uptake of Ca^2+^ was facilitated predominantly by SERCA. Sodium azide, a potent mitochondria inhibitor^46^ did not affect Ca^2+^ uptake, suggesting a lack of mitochondria-mediated Ca^2+^ uptake in this assay (see Supplementary Fig S3).

### Protein analysis

The protein expression of total cTnI, phosphorylated cTnI at Ser23/24, SERCA2a, total PLB, phosphorylated PLB at Ser16 and Thr17 and NCX1 was determined as described previously^13^. Briefly, protein homogenates from left ventricular tissue were separated on either 10% or 15% SDS–polyacrylamide gels depending on the protein molecular weight, and transferred onto nitrocellulose membranes. The membranes were blocked with 5% (w/v) skimmed milk and probed with specific antibodies for total cTnI (rabbit polyclonal, 1:1000 dilution in TTBS, Cell Signaling Technology), phosphorylated cTnI Ser23/24 (rabbit polyclonal, 1:1000 dilution in TTBS, Cell Signaling Technology),SERCA2a (rabbit polyclonal, 1:10,000 dilution in 5% Tween 20 Tris Buffered Saline (TTBS), Badrilla Ltd), total PLB (mouse monoclonal, 1:20,000 dilution in TTBS, Badrilla Ltd), phosphorylated PLB Ser16 (rabbit polyclonal, 1:5000 dilution in TTBS, Badrilla Ltd), phosphorylated PLB Thr17 (rabbit polyclonal, 1:5000 dilution in TTBS, Badrilla Ltd), NCX1 (rabbit monoclonal, 1:1000 dilution in TTBS, Abcam), or GAPDH (mouse monoclonal, 1:20,000 dilution in TTBS, GeneTex), with corresponding horseradish peroxidase-conjugated secondary antibodies (Abcam, Sapphire Bioscience Pty. Ltd). Proteins were visualised using an enhanced chemiluminescence detection system (SUPERSIGNAL West Pico PLUS Chemiluminescent Substrate, Thermo Scientific). The protein expression of total cTnI, SERCA2a, total PLB and NCX1 was normalised to GAPDH to correct for protein loading, whereas the phosphorylated proteins were normalised to the corresponding total proteins. Although some studies have suggested an alteration in GAPDH protein expression in diabetes^47,48^, others^16^ and our previous data^13,14^ did not find any change.

### Statistical analysis

Statistical analyses were performed using GraphPad Prism v.8.3.0. D’Agostino & Pearson test and Saphiro-Wilk test were used to check for a normal distribution. Student’s unpaired *t*-tests were used to test for differences between non-diabetic and diabetic groups. Results are presented as means ± SEM, a value of *P* < 0.05 was considered statistically significant.

### Ethical approval

All experiments and the use of animals were approved (AUP-19-179) and conducted in accordance with the guidelines of the Animal Ethics Committee of the University of Otago and in adherence to the NZ Animal Welfare Act 1999. All experiments were performed in compliance with the ARRIVE guidelines.

## Supplementary Information

Supplementary Figures.

## Data Availability

The datasets generated and/or analysed during the current study are available from the corresponding author upon reasonable request.
