# Supplementary material for: Sarco/endoplasmic reticulum calcium ATPase activity is unchanged despite increased myofilament calcium sensitivity in Zucker type 2 diabetic fatty rat heart
Source: Sci Rep. 2022 Oct 7;12:16904. doi: 10.1038/s41598-022-20520-0 (PMC9546843; doi:10.1038/s41598-022-20520-0)
Supplement: Supplementary file 1 — Supplementary Figures. [file 41598_2022_20520_MOESM1_ESM.pdf]

Supplementary information

Sarco/endoplasmic reticulum calcium ATPase activity is unchanged despite increased myofilament calcium sensitivity in Zucker type 2 diabetic fatty rat heart

Yann Huey Ng[1,2], Regis R. Lamberts[2], Peter P. Jones[2], Ivan A. Sammut[3], Gary M. Diffee[4], Gerard T. Wilkins[1], James C. Baldi[1,*]

[1]Department of Medicine and HeartOtago, Otago Medical School, University of Otago, Dunedin, New Zealand

[2]Department of Physiology and HeartOtago, School of Biomedical Sciences, University of Otago, Dunedin, New Zealand

[3]Department of Pharmacology and Toxicology and HeartOtago, School of Biomedical Sciences, University of Otago, Dunedin, New Zealand

[4]Department of Kinesiology, University of Wisconsin-Madison, Madison, WI, USA

*Corresponding author: chris.baldi@otago.ac.nz

To whom correspondence should be addressed:

Associate Professor James C. Baldi, Department of Medicine and HeartOtago, Otago Medical School, University of Otago, 201 Great King Street, Dunedin Central, Dunedin 9016, New Zealand. Tel: +64 3 470 9991, Email: chris.baldi@otago.ac.nz

Supplementary figures

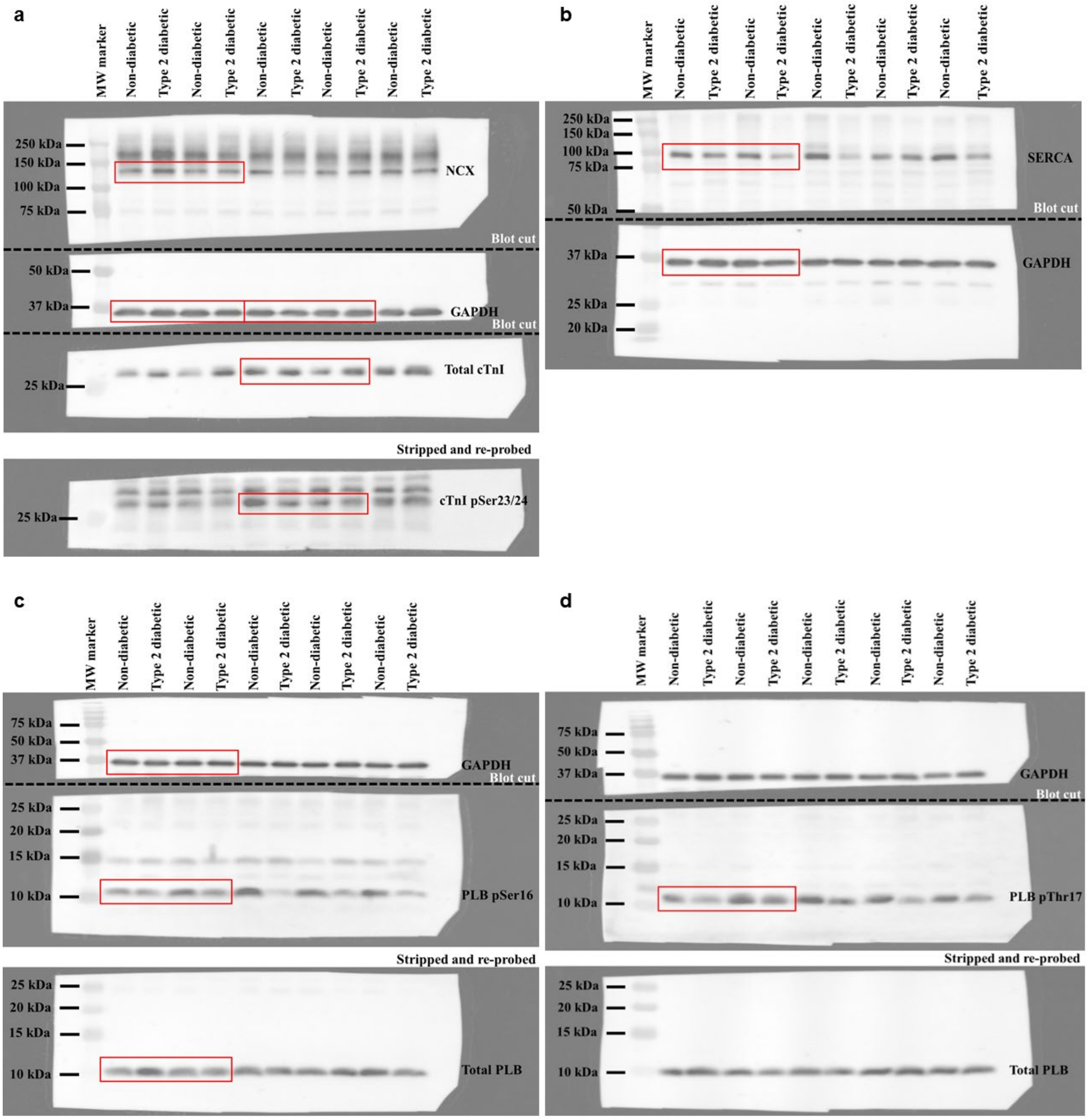

Figure S1. Original uncropped immunoblots. Representative immunoblots for (a) NCX, total cTnI and cTnI pSer23/24, (b) SERCA, (c) total PLB and PLB pSer16, and (d) total PLB and PLB pThr17 in non-diabetic and diabetic rat left ventricular tissues. NCX and cTnI were run on the same blot (panel a). Phosphorylated PLB at Ser16 (panel c) and Thr17 (panel d) were run on separate blots to prevent excessive stripping of the membranes, and normalised to the respective total PLB probed on the same blot. Red boxes indicate regions that were cropped and presented in figures within the manuscript. Membranes in all panels were cut as indicated by the black dotted lines to assess multiple proteins on the same blot. MW, molecular weight.

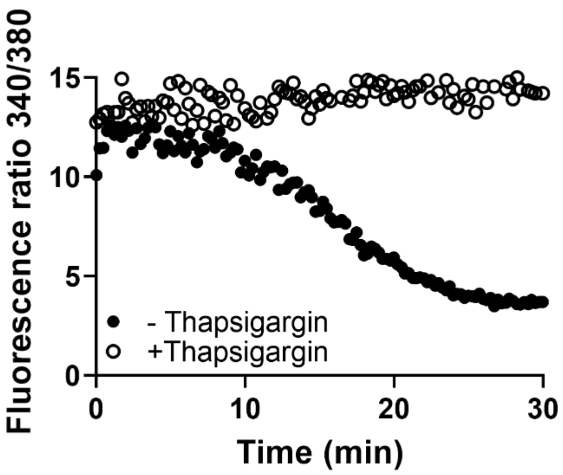

Figure S2. Inhibition of SERCA Ca^{2+} uptake in the presence of thapsigargin. A representative fura-2 fluorescence ratio 340/380 *versus* time curves in isolated rat cardiac sarcoplasmic reticulum vesicles in the absence (-Thapsigargin) or presence (+Thapsigargin) of thapsigargin, confirming SERCA-mediated Ca^{2+} uptake.

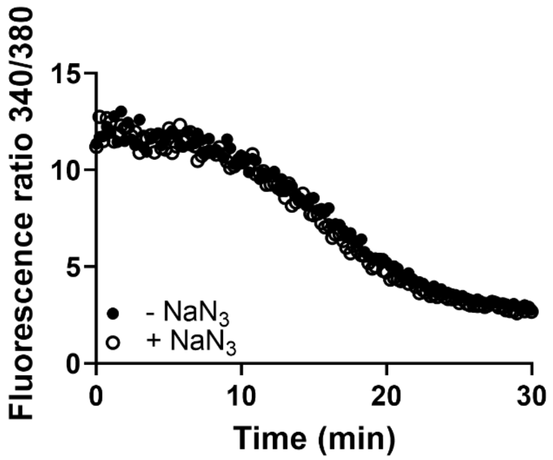

Figure S3. SERCA Ca^{2+} uptake was not affected in the presence or absence of sodium azide. A representative fura-2 fluorescence ratio 340/380 *versus* time curves in isolated rat cardiac sarcoplasmic reticulum vesicles in the absence (-NaN_3) or presence (+NaN_3) of sodium azide, confirming that mitochondria was not involved in mediating Ca^{2+} uptake in this assay.
